# Genetic and epigenetic analysis of schizophrenia in blood—a no-brainer?

**DOI:** 10.1186/s13073-016-0354-4

**Published:** 2016-09-21

**Authors:** Andrew E. Jaffe, Joel E. Kleinman

**Affiliations:** 1 Lieber Institute for Brain Development, Johns Hopkins Medical Campus, Baltimore, MD 21205 USA; 2 Department of Mental Health, Johns Hopkins Bloomberg School of Public Health, Baltimore, MD 21205 USA; 3 Department of Biostatistics, Johns Hopkins Bloomberg School of Public Health, Baltimore, MD 21205 USA; 4 Center for Computational Biology, Johns Hopkins University, Baltimore, MD 21205 USA; 5 Department of Psychiatry and Behavioral Sciences, Johns Hopkins School of Medicine, Baltimore, MD 21205 USA

## Abstract

Several recent studies have investigated either genetic or epigenetic variation in schizophrenia. A recent study presents comprehensive analyses of blood samples to better characterize the combined role of genetic and epigenetic variation in schizophrenia. While the paper identifies significant associations with schizophrenia risk and diagnosis, the potential relevance to the brain in schizophrenia is questionable.

## Characterizing genetic and epigenetic variation in schizophrenia

The latest genome-wide association study (GWAS) for schizophrenia has identified 108 genetic loci that significantly confer risk for schizophrenia, with common variants explaining 18.4 % of the overall variation in diagnosis [1]. How genetic variation influences risk for schizophrenia has been a question of great importance to the field and recent research has suggested that DNA methylation (DNAm) may mediate genetic risk for a large number of the GWAS loci in the human adult [2] and developing [3] brain. However, brain tissue is difficult to collect and assay in the large number of samples that are probably necessary to see more subtle effects of genetic risk on local epigenetic variation and thus researchers often use blood as a surrogate tissue. There are still many lingering questions surrounding how epigenetic changes in the blood associated with exposure or outcome relate to changes in other tissues, particularly the brain.

Recently, Hannon and colleagues [4] have presented comprehensive analyses of large blood DNAm datasets to better characterize the combined role of genetic and epigenetic variation in schizophrenia. In this study, using microarrays designed to measure DNAm across the epigenome, the authors first identified sites (individual microarray probes) and regions (groups of probes) differentially methylated between patients with schizophrenia and unaffected controls in blood. These differentially methylated loci related to genomic regions involved in immune function, neuronal proliferation, and brain development and also significantly overlapped with the 108 GWAS risk loci previously identified for schizophrenia [1]. By integrating genetic information on the same subjects, Hannon and colleagues [4] identified sites in the epigenome associated with overall genetic risk for schizophrenia (polygenic risk scores (PRSs)). Interestingly, there was little overlap between the effects of PRS levels (those sites related to genetic risk) and differentially methylated loci (those sites perhaps related to the consequences of illness) between patients and controls (Fig. 3 in [4]). The authors also performed Bayesian co-localization analyses combining GWAS summary statistics and methylation quantitative trait loci (mQTLs) and provided evidence of convergence of genetic and epigenetic risk for schizophrenia. A subset of previously identified co-localized signals in the brain [3] was also identified in the Hannon et al. [4] blood dataset (43.6 %, 7/16), including three mapping to the *AS3MT* locus that have already been linked to a variable number of tandem repeats (VNTR) polymorphism in that locus that is considered to be a likely causal genetic variant for schizophrenia [5].

## Clinical and molecular heterogeneity underlies schizophrenia

This work joins several other recent large-scale, multi-phase studies characterizing DNAm changes related to case–control differences in schizophrenia in the blood, including Aberg and co-workers [6] and Montano and co-workers [7]. While all three studies each implemented stringent statistical analysis and independent replication, there was surprisingly little overlap of differential methylation signals across the studies (Table 1). For example, none of 25 significantly differentially methylated sites/probes identified by Hannon and colleagues [4] in the discovery sample (at *p* < 1 × 10^−7^; Table 1 in [4]) were present in either previous study (Table 1), including the 172 significant and replicated probes and nearby genes from Montano et al. [7] (eTable 4 in [7]) or the 65 unique genes with differential methylation signal reported by Aberg et al. [6] (Table 2 in [6]). Even among the 1223 marginally significant (*p* < 1 × 10^−5^) probes identified by Hannon and colleagues [4] in the discovery sample (Table S3 in [4]) there was minimal overlap in differential methylation signal at the probe (*N* = 2) or gene (*N* = 9 and *N* = 3, respectively) levels. As there was only one gene in common (*SATB1*) between the previous two studies, these three studies largely present non-overlapping regions of the epigenome associated with schizophrenia and the plethora of epiphenomena that accompany the disorder. Together, these studies underscore the extensive clinical and molecular heterogeneity underlying this debilitating brain disorder.

**Table 1:** Replication of significant case–control differences

Discovery threshold	Type	Study	Tissue	Probes^a^	Genes^b^
*p* < 1 × 10^−7^	Discovery	Hannon et al. [4] (Table 1)	Blood	25	16
	Replication	Montano et al. [7] (eTable 4, *N* = 172)	Blood	0	0
		Aberg et al. [6] (Table 2, *N* = 65)	Blood	NA	0
		Jaffe et al. [2] (Table S10, *N* = 2,104)	Brain	0	1
*p* < 1 × 10^−5^	Discovery	Hannon et al. [4] (Table 1)	Blood	1223	949
	Replication	Montano et al. [7] (eTable 4, *N* = 172)	Blood	2	9
		Aberg et al. [6] (Table 2, *N* = 65)	Blood	NA	3
		Jaffe et al. [2] (Table S10, *N* = 2,104)	Brain	6	61

We examined the sites identified as differentially methylated in the Hannon et al. [4] study in the context of previous work across other large blood and brain datasets

^a^Probes refer to the exact Illumina 450 k probe by “cg” identifier

^b^Genes refer to the nearest gene to each probe or those listed in the tables of the cited papers

## Outstanding questions and next steps

As compelling as these findings are, several questions still remain regarding the relevance to the brain—the tissue most relevant to the etiology of schizophrenia—and the residual influence on epigenetics of other factors such as cellular composition, antipsychotic medication, substance abuse, and stress.

For both the case–control differences and also the co-localization of mQTLs and schizophrenia GWAS risk single nucleotide polymorphisms (SNPs), we would be interested in what proportion of blood-identified signal replicates in brain samples. While Hannon and colleagues [4] ask the reverse question—what proportion of a lesser number of brain-identified co-localization signals were present in the blood—we believe that the former question would more directly highlight the usefulness of blood as a surrogate for better understanding the causes and consequences of schizophrenia. We further found little overlap between probes differentially methylated in the present study and those previously identified as differentially methylated between patients and controls in the frontal cortex (6/1223 probes; Table 1) [2].

As mentioned, another question relates to potential residual influences of cellular composition, medications, substance abuse, and stress in the case–control differences identified in blood. While the authors statistically adjusted for the effect of cellular composition, a very strong potential confounder in epigenome-wide association studies (EWAS) [8] and smoking, one of the strongest environmental influences on DNAm in blood [9], these other potential confounders might similarly have strong influences on DNAm but could be much harder to quantify due to the large number of potential medications used for treatment, the large number of substances abused by patients, and the nature of stress. Furthermore, the heterogeneous collection of cell types in the blood, each with unique epigenetic signatures and with potentially variable ages and lifespans of cells since leaving the bone marrow, makes whole blood a difficult tissue to interpret epigenetic differences.

Ultimately, this study pushes the boundaries of EWAS of schizophrenia in the blood and reminds us of the importance of studying the brain in schizophrenia in future work. While blood may be a useful biomarker, identifying epigenetic factors and their interplay with genetic risk variants in the brain offers the promise of finding novel targets and treatments to improve the lives of patients with schizophrenia.

## Abbreviations

DNAm: DNA methylation
EWAS: epigenome-wide association studies
GWAS: genome-wide association studies
mQTL: methylation quantitative trait loci
PRS: polygenic risk score
SNP: single nucleotide polymorphism
VNTR: variable number of tandem repeats
